# The effect of *Urtica dioica* extract on oxidative stress, heat shock proteins, and brain histopathology in multiple sclerosis model

**DOI:** 10.14814/phy2.15404

**Published:** 2022-08-03

**Authors:** Fatemeh Namazi, Elnaz Bordbar, Farnoosh Bakhshaei, Saeed Nazifi

**Affiliations:** ^1^ Pathology Division, Department of Pathobiology School of Veterinary Medicine, Shiraz University Shiraz Iran; ^2^ School of Veterinary Medicine Shiraz University Shiraz Iran; ^3^ Clinical Pathology Division, Department of Clinical Sciences School of Veterinary Medicine, Shiraz University Shiraz Iran

**Keywords:** heat shock protein, histopathology, multiple sclerosis, *Urtica dioica*

## Abstract

Multiple sclerosis (MS) results from the destruction of myelin and focal inflammation. The study aimed to evaluate the effect of hydroalcoholic extract of *Urtica dioica* on oxidative stress, heat shock proteins, and brain histopathology in multiple sclerosis model. Sixty male C57BL/6 mice were divided into six groups of 10. Groups included positive control, negative control, and treatment groups with *U. dioica* extract at a dose of 50, 100, 200, and 400 mg/kg for 21 days (three times a week). The MS model was developed by a diet containing 0.2% cuprizone for 6 weeks. A section of brains was evaluated with Luxol Fast Blue staining and the other part evaluated with heat shock protein (HSP) kits 60 and 70, total antioxidant capacity (TAC), and malondialdehyde (MDA). In sections of corpus callosum, the highest amount of myelin was observed in the negative controls, while the use of cuprizone in the positive controls caused the destruction and reduction of myelin. The use of *U. dioica* extract in therapeutic groups except at a dose of 50 mg/kg could reduce myelin degradation to some extent and lead to remyelination. However, myelin levels in treatment groups were not significantly different from any of the negative and positive controls. Although HSP60 decreased in the treatment groups, there was no significant difference between the positive and negative controls. Treatment with this extract significantly reduced the amount of HSP70 compared with the positive controls. The decreased TAC and increased MDA in positive controls indicated oxidative stress, respectively. Furthermore, the extract led to an increase and decrease of TAC and MDA in the treatment groups, respectively. However, only the MDA level was significantly different from that of the positive controls. Therefore, the antioxidant effects of *U. dioica* extract could decrease cuprizone‐induced oxidative stress and be effective in improving demyelination.

## Graphical abstract

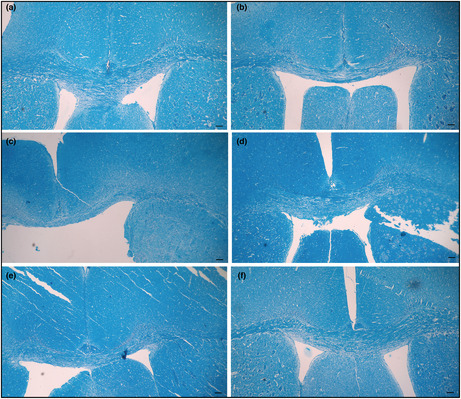

## 1 INTRODUCTION

Multiple sclerosis (MS) is a chronic, progressive neurodegenerative disease. Demyelination in white and gray matter, loss of oligodendrocytes and axons, and activation of microglia are histopathological characteristics of MS (Goldberg et al., 2015). Interaction of brain resident cells (such as microglia and astrocytes) and peripheral immune cells (such as lymphocytes and monocytes) results in demyelination (Carassiti et al., 2018). With regard to the spatial distribution of the lesions in the central nervous system (CNS), varied neurological symptoms occur in patients. The most common symptoms of the disease include sensory problems, cognitive dysfunction, dizziness and vertigo, unilateral painless loss of vision, double vision, motor weakness, ataxia, fatigue, and bowel and bladder troubles (Huang et al., 2017). Oxidative stress plays a role in brain damage. Reactive oxygen species (ROSs) contribute to the pathogenesis of MS lesions and the progression of demyelination through different mechanisms (Adamczyk & Adamczyk‐Sowa, 2016; Ortiz et al., 2013). Despite the key roles of ROSs as regulatory mediators in signaling pathways and the stability of neurons, there is a fine line between the necessity and harmfulness of ROSs (Padureanu et al., 2019; Pegoretti et al., 2020). Malondialdehyde is produced by lipid peroxidation and is widely used as a biomarker to determine oxidative stress levels (Haritha & Sangeetha, 2020). In response to stressors such as inflammation, heat stress, hypoxia, and oxidative damage, cells upregulate the expression of their heat shock proteins to promote their survival (Cwiklinska et al., 2020; Gorter et al., 2018). Heat‐shock proteins (HSPs) are classified as chaperones in different families by their molecular mass. These molecules play a role in properly folding newly‐synthesized proteins and proteins exposed to stress‐induced denaturation (Turturici et al., 2014).

*Urtica dioica* (*U. dioica*) belongs to the family Urticaceae and is an annual and perennial plant (Loshali et al., 2019) known as “Gazaneh” in Iran. This plant contains minerals, chlorophyll, amino acids, lecithin, carotenoids, and vitamins; thus, it is consumed as one of the important medicinal plants (Asgarpanah & Mohajerani, 2012) and has a strong antioxidant capacity (Bourgeois et al., 2016; Khare et al., 2012).

The toxin models of multiple sclerosis allow studying specific aspects of the disease such as demyelination and remyelination. Cuprizone [oxalic acid bis (cyclohexylidenehydrazide)] is a copper‐chelating factor that leads to demyelination within the central nervous system (Pandur et al., 2019). The cuprizone model is suitable and well‐known for studying the potency of the compound to regulate myelination (Torkildsen et al., 2008; Zhan et al., 2020).

The present study investigated the potential of *U. dioica* to inhibit cuprizone‐induced demyelination or accelerate remyelination in a mice model and examined the changes of total antioxidant capacity, malondialdehyde, and heat shock proteins70 and 60 in brain tissue in control and treatment group.

## 2 MATERIALS AND METHODS

### 2.1 Extract

The plant was purchased from an herbal pharmacy, Shiraz, Iran. Aerial parts of the plant were washed, air‐dried at room temperature, and grounded into fine mass. 50 g powder of sample was soaked in 500 ml of 70% ethanol for 24 h, and then filtered through Whatman Grade No.2 paper. The hydroalcoholic extract was evaporated in a rotary evaporator at 50°C under vacuum (3 h) to solvent removal. Subsequently, it was lyophilized and stored at −20°C until further use.

### 2.2 Animals

Eight‐week‐old male C57BL/6 mice weighing 19–21 g were obtained from Pasteur Institute of Tehran, Iran. Mice were acclimatized to the laboratory environment at 22–25°C under 12‐h light and dark cycle with free access to tap water and food (commercial chow) for 1 week. This setting was maintained during the study.

### 2.3 Experimental design

Animals were divided into six groups of 10, including four treatment groups, one positive control group, and one negative control group. To induce demyelination, all groups, except the negative control group, were fed standard chow containing 0.2% cuprizone (0.2 g per 100 g of food) for 6 weeks. The negative control group was fed standard chow. Four treatment groups received *U. dioica* extract in four doses (50, 100, 200, and 400 mg/kg) by oral gavage three times a week for 21 days after induction of demyelination (end of the sixth week). Negative control received distilled water by oral gavage three times a week for 21 days and positive control received no treatment after inducing demyelination.

### 2.4 Preparation of tissue samples

After three weeks of *U. dioica* treatment, the mice were euthanized by ether and a part of the brain were excised, and stored at −20°C until HSPs, TAC, and MDA determination. Another part of the brain was fixed in 10% neutral buffered formalin, embedded in paraffin, and used for pathological studies. Tissue sections were stained with Luxol Fast Blue staining and investigated by light microscopy as previously described (Acs et al., 2009). The area of the corpus callosum was then assessed by Digimizer software.

### 2.5 Preparation of tissue extracts

Five hundred (500) mg of the brain tissue was crushed and sodium phosphate buffer (0.1 M, pH = 7.4) was added 5 times its weight and then homogenized with a homogenizer. The suspensions were centrifuged for 15 min at 750*g* and 4°C. The supernatants were transferred to 0.5 ml microtubes and placed at −20°C.

### 2.6 Determination of HSPs

Tissue levels of HSP70 and HSP60 were measured by a quantitative sandwich enzyme immunoassay using commercial rat‐specific kits (Shanghai Crystal Day Biotech, Shanghai, China). The sensitivity of the HSP70 kit was 0.021 ng/ml. The intra‐assay precision and inter‐assay precision of the HSP 70 kit were CV < 8% and CV < 10%, respectively. The sensitivity of the HSP60 kit was 0.023 ng/ml. The intra‐assay precision and inter‐assay precision of HSP60 kit were CV < 8% and CV < 10%, respectively.

### 2.7 Oxidative stress index assay

A commercial kit (ZellBio GmbH kit) was used to determine the TAC level. The color product of the chromogenic substrate (tetramethylbenzidine) emerged at the ending phase. The difference in color was calculated calorimetrically using a spectrophotometer (Jenway 6300 Spectrophotometer) at 450 nm and represented as mmol/L. This method can determine TAC with 0.1 mM sensitivity (100 μmol/L). The intra‐ and inter‐assay CVs were below 3.4% and 4.2%, respectively.

An assay kit purchased from ZellBio GmbH was used to measure MDA (μmol/L; Cat. no. ZB‐MDA96A). In this kit, MDA is measured based on its reaction with thiobarbituric acid in an acidic condition and high temperature. The color complex was measured colorimetrically at 535 nm. The assay kit sensitivity was 0.1 μM (inter‐assay CV: 5.8%) for MDA.

### 2.8 Statistical analysis

SPSS software version 16 (SPSS Inc.) was applied to analyze data, and Graphpad Prism version 8.0.2 (GraphPad Software Inc.) was used to draw graphs. The data were analyzed by one‐way ANOVA followed by Tukey's test. The data were shown as mean ± SE, and *p* < 0.05 was regarded as significant.

## 3 RESULTS

**FIGURE 1:**
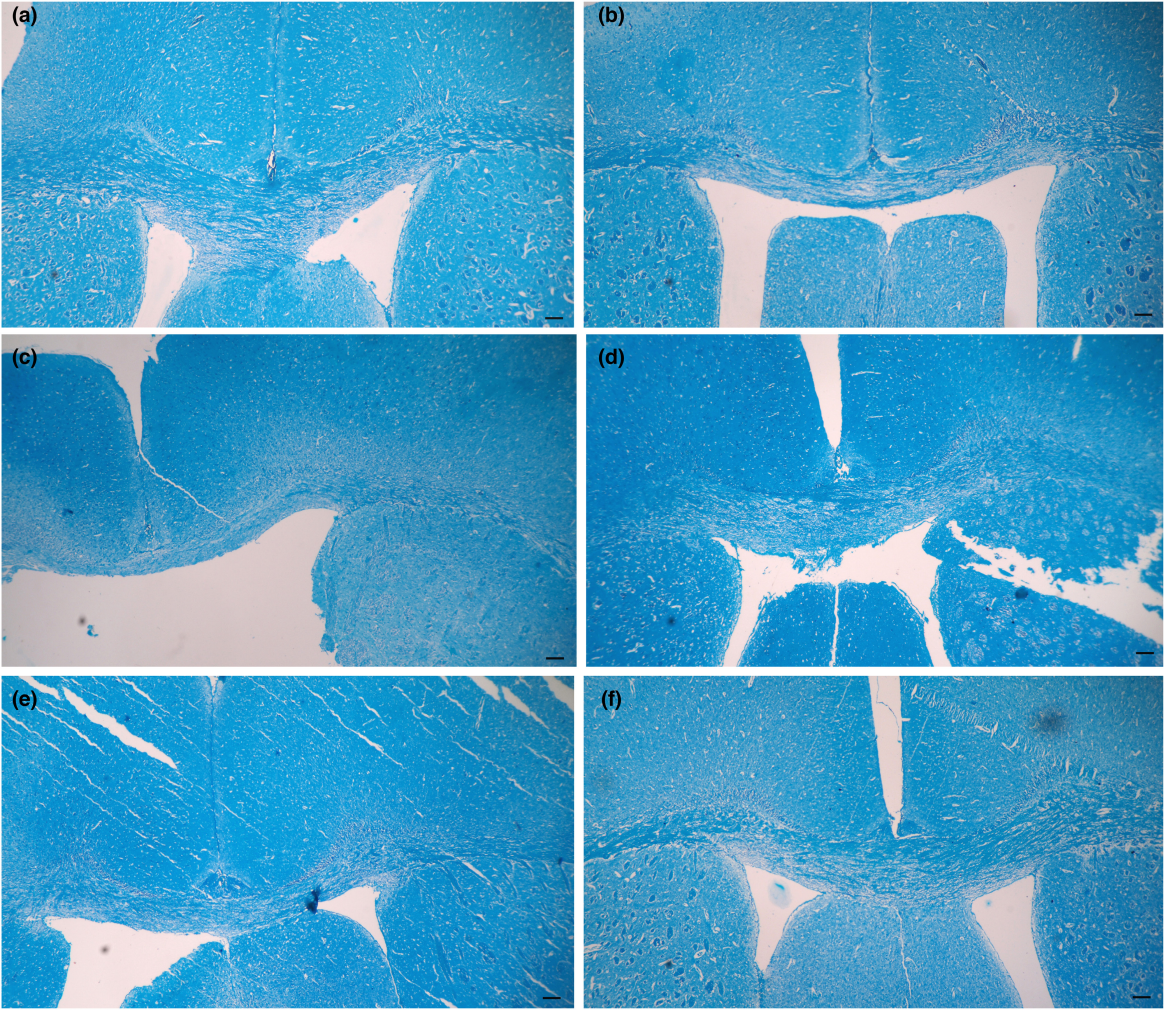
Evaluation of corpus callosum with Luxol fast blue staining. (a) Negative control group with the highest amount of myelin. (b) Positive control group with decrease of myelin. (c) Treatment with 50 mg/kg of *U. dioica* extract. (d) Treatment with 100 mg/kg of *U. dioica* extract. (e) Treatment with 200 mg/kg of *U. dioica* extract. (f) Treatment with 400 mg/kg of *U. dioica* extract. Among treated groups, the highest amount of myelin was observed in mice received 400 mg/kg dose. Scale bar = 250 μm.

**FIGURE 2:**
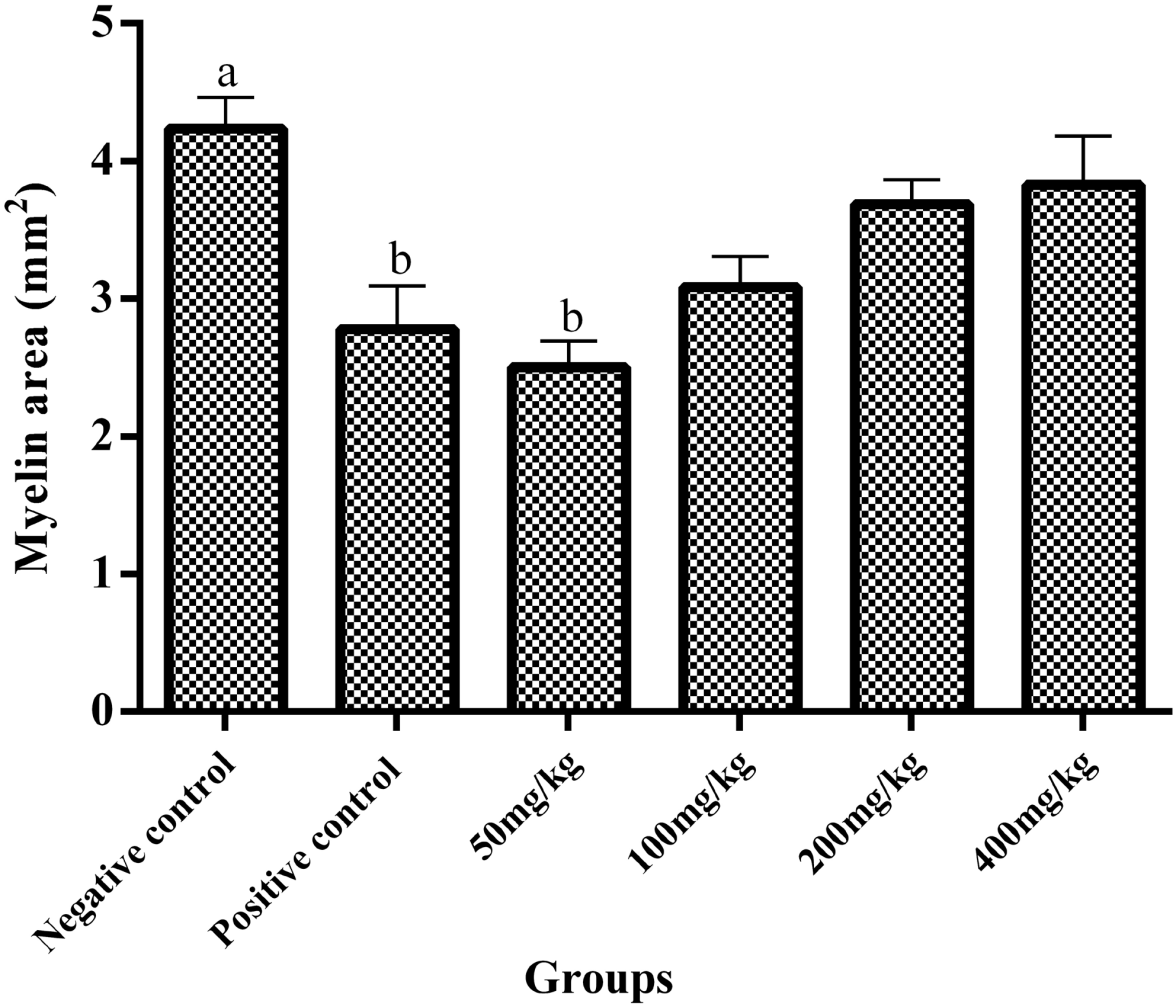
Comparison of the amount of myelin in the corpus callosum in different groups (mean ± SE). The consumption of *U. dioica* extract in therapeutic groups except for 50 mg/kg dose reduced myelin degradation, though there was no significant difference in the amount of myelin in treatment groups with any of the negative and positive control groups. (a) Significantly different from positive group at *p* < 0.05. (b) Significantly different from negative group at *p* < 0.05.

In the histopathological evaluation of corpus callosum with Luxol Fast Blue staining, the highest amount of myelin was observed in the negative control group (4.23 ± 0.22 mm^2^), while the use of cuprizone in the positive control group caused myelin destruction (2.77 ± 0.31 mm^2^). There was statistically a significant difference between the positive and negative control groups (*p* = 0.00). The results of the present study showed that consumption of *U. dioica* extract in therapeutic groups except for 50 mg/kg dose reduced myelin degradation and accelerated remyelination. As among treated groups, the highest amount of myelin was observed in mice received 400 mg/kg dose of *U. dioica* extract. However, the results showed an insignificant difference in the amount of myelin in treatment groups with any of the negative control and positive control groups (Figures 1 and 2).

Effect of different doses of *U. dioica* on total antioxidant capacity, malondialdehyde, and heat shock proteins 70 and 60 in brain tissue was presented in Table 1 (Mean ± SE). HSP70 levels showed a significant elevation in the positive control group compared with the negative control group (*p* = 0.02). HSP70 levels decreased significantly in all therapeutic groups except 50 mg/kg dose compared with the positive control (group 50 mg/kg: *p* = 0.18, group 100 mg/kg: *p* = 0.00, group 200 mg/kg: *p* = 0.05, group 400 mg/kg: *p* = 0.05), and showed no significant difference as compared with the negative control group. HSP60 levels in the positive control group increased compared with the negative control group, but this increase was not statistically significant (*p* = 0.56). Although the levels of HSP60 in the *Urtica dioica*‐treated groups were lower than the positive control group, no significant differences were observed between the groups.

**TABLE 1:** Effect of different doses of *U. dioica* on heat shock proteins70 and 60, malondialdehyde and total antioxidant capacity in brain tissue of acute cuprizone‐induced demyelination mice models (mean ± SE)

Group	HPS60 (ng/ml)	HPS70 (ng/ml)	MDA (μmol/L)	TAC (μmol/L)
Positive control	7.94 ± 0.19	105.00 ± 23.25^a^	41.59 ± 4.66^a^	7.41 ± 1.28
Negative control	6.68 ± 0.57	51.28 ± 6.46^b^	13.89 ± 2.12^b^	11.66 ± 0.65
Treatment with 50 mg/kg of *U. dioica* extract	7.19 ± 0.45	67.55 ± 8.33	20.51 ± 4.47^b^	9.51 ± 2.18
Treatment with 100 mg/kg of *U. dioica* extract	7.02 ± 0.41	37.37 ± 5.36^b^	10.96 ± 2.18^b^	9.92 ± 0.61
Treatment with 200 mg/kg of *U. dioica* extract	7.74 ± 0.53	58.66 ± 6.53^b^	13.06 ± 1.60^b^	22.95 ± 7.19^b^
Treatment with 400 mg/kg of *U. dioica* extract	6.43 ± 0.52	58.50 ± 12.16^b^	8.01 ± 1.24^b^	11.08 ± 1.06

*Note*: Data are presented as means ± SE.

Abbreviations: HSP, Heat shock protein; MDA, Malondialdehyde; TAC, Total antioxidant capacity.

^a^ Significantly different from negative group at *p* < 0.05.

^b^ Significantly different from positive group at *p* < 0.05.

The brain tissues of the positive control group had lower levels of TAC compared with the negative control group, but this difference was insignificant (*p* = 0.96). In therapeutic groups, although TAC levels increased compared with the positive controls, there was no significant difference with positive and negative control groups. Increased TAC in the group treated with 200 mg/kg dose of *U. dioica* extract showed a significant difference with positive control group (*p* = 0.04). MDA levels were significantly lower in the negative control compared with positive control (*p* = 0.00). Using the extract decreased significantly the MDA levels in treated mice in comparison to the positive control (*p* = 0.00). No significant difference was seen in therapeutic groups with negative controls.

## 4 DISCUSSION

Cuprizone is an experimental model to evaluate multiple sclerosis. As in the present study, demyelination was observed in the corpus callosum following its consumption. Cuprizone as well as MS targets mature oligodendrocytes (Dutta & Trapp, 2014; Montes et al., 2014), which causes demyelination and depletion of oligodendrocytes in various areas of the brain including corpus callosum (Koutsoudaki et al., 2009). Furthermore, oxidative stress often results in oligodendrocyte apoptosis and the death of the neuronal cell (Kipp et al., 2009). It has been concerned that cuprizone induces damage through excess production of free radicals, and decrease of superoxide dismutase (SOD) and glutathione peroxidase (GPx) levels (Ghaiad et al., 2017; Praet et al., 2014; Trapp & Nave, 2008). Different studies indicated that different herbal medicines like *Curcuma longa* and *Nigella sativa* enhance remyelination in CNS (Mojaverrostami et al., 2018). In this study, the histopathological evaluation of tissue sections revealed an increased amount of myelin in treatment groups compared with the positive control group, though there was no significant difference between any of the negative and positive control groups.

Various studies showed the effect of oxidative stress on MS. The CNS due to several factors such as high energy demand and mitochondrial activity, limited cell renewal, and high levels of iron and unsaturated fatty acids is highly susceptible to oxidative stress. In active MS lesions, oligodendrocytes have been shown to contain high levels of oxidized DNA, and oxidized phospholipids predominantly accumulate in axons with disrupted transport. Also, the severity of oxidative damage appears to be related to the degree of inflammation (Pegoretti et al., 2020). On the other hand, antioxidants as preventive and therapeutic agents are able to eliminate free radicals and reactive oxygen species. It has been reported that D‐aspartic acid consumption increased TAC and decreased pathological lesions in experimental autoimmune encephalomyelitis (Afraei et al., 2017). Antioxidant and anti‐inflammatory effects of Ginseng has evaluated on MS, which caused the reduction of demyelination and an increase in immune cell activity (Lee et al., 2019).

Studies have revealed the antioxidant properties of *U. dioica* hydroalcoholic extract (Mavi et al., 2004). A study on the effects of the hydroalcoholic extract of *U. dioica* on oxidative stress in patients with type 2 diabetes reported increases in TAC and SOD and no changes in MDA and glutathione peroxidase (Namazi et al., 2012). Cetinus et al. (2005) found *U. dioica* to decrease tourniquet‐induced oxidative stress in the ischemic muscle of rats by decreasing MDA levels (Cetinus et al., 2005). Moreover, 400 mg/kg of *U. dioica* extract was found to exert protective effects on the liver by decreasing MDA and increasing SOD (Sarma Kataki et al., 2012). In the present study, treatment with *U. dioica* extract increased the total antioxidant capacity compared with the positive control group, although the changes were not statistically significant. In addition, using the extract decreased MDA levels compared with the positive control group and changes were statistically significant.

Many properties of plants such as antioxidant, hypoglycemia, anti‐inflammatory, and antivirus are related to acid chlorogenic (Alonso‐Castro et al., 2008; Dos Santos et al., 2006). Acid chlorogenic has been reported as the most phenolic compound in *U. dioica* extract, and is responsible for its anti‐inflammatory and antinociceptive activities (Marrassini et al., 2010). Therefore, it seems that antioxidant compounds, as free radical scavengers, are essential for sustaining the function of oligodendroglia.

Heat shock protein 70 protects cells against stress‐induced lethal damage and plays a key role in immune responses (Pockley et al., 2014; Radons, 2016). Given the reported anti‐inflammatory effects of HSP70 on MS lesions, its over‐expression can help repair myelin and protect tissues against demyelination; nevertheless, the exact role of HSP70 in MS requires further investigations (Mansilla et al., 2012). It is noteworthy that extra‐cellular HSP molecules are functionally different from their intra‐cellular counterparts. Intracellular HSPs play protective roles, whereas extracellular HSP molecules create responses in innate and adaptive immune systems (Asea et al., 2017). Phylogenetic protection, for example immune responses to bacterial HSPs and cross‐reaction with HSPs in mammal and their potential for stimulating strong immune responses enable HSPs to induce or continue autoimmune diseases (Turturici et al., 2014). According to Cwiklinska et al., the inhibition of HSP70 reduces the expression of Th17 genes and alleviates autoimmune demyelination. They also found HSP70 to facilitate the specific miRNA function that causes the expression of Th17 genes (Cwiklinska et al., 2020). Measuring HSP70 in the present study showed elevated brain levels of HSP70 through cuprizone consumption, which was significant in the positive control compared with the negative control group. Except for 50 mg/kg, all doses of *U. dioica* extract significantly decreased HSP70 levels compared with the positive control group.

It seems that HSP60 plays a key role in the immune system and intercedes immune physiology (Quintana & Cohen, 2011). It has been reported knockdown of HSP60 led to the decline of inflammation in the Japanese encephalitis virus (Swaroop et al., 2018). In addition, HSP60 can contribute to neuroinflammation by interacting with TLR4 at the microglial surface (Lehnardt et al., 2008) and inducing the generation of pro‐inflammatory agents (Zhang et al., 2012). The intrathecal injection of HSP60 caused nerve destruction and demyelination by activating TLR4‐MyD88 signaling in microglial cells (Rosenberger et al., 2015). In this study, the increase in HSP60 levels was statistically insignificant in the positive control compared with the negative control group. Treating with *U. dioica* extract slightly decreased this factor, though there are no significant differences compared with positive and negative control groups. Treatment with *U. dioica* extract may reduce extra‐cellular HSP molecules and their role in autoimmune disease, but this hypothesis requires further investigation.

The antioxidant properties of *U. dioica* extract appear to reduce the effects of cuprizone‐induced oxidative stress and the plant might be effective to treat and mitigate demyelination. In line with previous studies, the present research found *U. dioica* extract to decrease HSP70 and HSP60 and alleviate demyelination; nevertheless, it is recommended that further studies be conducted to investigate the role of heat shock proteins.

## AUTHOR CONTRIBUTIONS

Fatemeh Namazi contributed to the study design; Fatemeh Namazi, Saeed Nazifi, and Elnaz Bordbar performed the study and analysis; and Fatemeh Namazi, Saeed Nazifi and Farnoosh Bakhshaei prepared the manuscript. All authors read and approved the final manuscript.

## FUNDING INFORMATION

This study was supported by the School of Veterinary Medicine, Shiraz University (grant number: 71‐GR‐VT‐5 and 99GCB1M83832) and Research Council of Shiraz University.

## CONFLICT OF INTEREST

The authors declare that they have no conflict of interest.

## ETHICAL STATEMENT

The procedures were in accordance with the guidelines of Shiraz University for the care and use of laboratory animals (IACUC no: 4687/63).
